# Nutritional and sensory quality of composite extruded complementary food

**DOI:** 10.1002/fsn3.940

**Published:** 2019-01-28

**Authors:** Sirawdink Fikreyesus Forsido, Haile Tesfaye Duguma, Tefera Belachew Lema, Barbara Sturm, Oliver Hensel

**Affiliations:** ^1^ Faculty of Organic Agricultural Sciences Department of Agricultural and Biosystems Engineering University of Kassel Witzenhausen Germany; ^2^ Department of Post‐Harvest Management College of Agriculture and Veterinary Medicine Jimma University Jimma Ethiopia; ^3^ Nutrition Unit Department of Population and Family Health College of Public Health and Medical Sciences Jimma University Jimma Ethiopia; ^4^ School of Agriculture, Food and Rural Development Newcastle University Newcastle upon Tyne UK

**Keywords:** blending ratio, cereal fortification, composite flour, food extrusion, food ingredients, sensory properties

## Abstract

Complementary foods in Ethiopia have nutritional and sensory limitations which can be attributed to cereal‐dominated ingredients and lack of appropriate processing techniques. This study aimed to optimize the nutritional and sensory quality of complementary food product through compositing and extrusion of various local ingredients. A constrained D‐optimal mixture experiment with 13 runs was designed. Accordingly, 55–65 g/100 g oats, 11–23 g/100 g soybean and 6–11 g/100 g linseed, and a premix of 9.9 g/100 g sugar, 0.6 g/100 g table salt, three g/100 g moringa and 1.5 g/100 g fenugreek were blended and extruded using a co‐rotating twin screw extruder with set parameters. Statistical model evaluation and optimization were done using Minitab version 16 software package. There is a statistically significant (*p* < 0.05) association between the blend of oats and soybean, oats and linseed, soybean and linseed, and the protein, fat, carbohydrate, fiber, ash, β‐carotene content as well as aroma, taste, and consistency. On the contrary, there is a no statistically significant (*p* < 0.05) association between the blends and moisture, energy, and zinc content together with appearance and overall acceptability. The optimal blending ratio was 55.0 g/100 g oats, 21.0 g/100 g soybean, and 9.0 g/100 g linseed plus 15.0 g/100 g premix. Evidence‐based selection of locally grown plant‐based ingredients, an optimal mixture of these ingredients and optimal processing, can result in a complementary food product with an improved dietary quality for children in low‐income settings.

## 1 INTRODUCTION

Child undernutrition in Ethiopia is associated with several diet‐related factors. First, there is inappropriate complementary feeding of children. A study has shown that only 4 percent of youngest children (6–23 months) living with their mothers in Ethiopia are fed per the national infant and young child feeding (IYCF) guidelines (Central Statistical Agency [Ethiopia] & ICF International, 2012). Second, traditional complementary foods are of low nutritional quality. A countrywide demographic survey showed the traditional complementary foods in Ethiopia are cereal‐based (e.g., Atmit) (69%), whereas consumption of vitamin A‐rich fruits and vegetables and foods made from roots and tubers are less common (24%–25%) (Central Statistical Agency, 2012). Third, nutrient and energy‐dense complementary foods are not affordable for many families in developing countries like Ethiopia (Muhimbula, Issa‐Zacharia, & Kinabo, 2011).

The final quality of traditional starchy complementary foods can be upgraded by carefully addressing the following three important factors (Muteki, MacGregor, & Ueda, 2007). The first factor is an optimal selection of raw materials. Low‐cost protein‐rich ingredients like legumes (also called pulses) are known to upgrade starchy foods (Egounlety, 2002; Mensah & Tomkins, 2003). Fat‐rich ingredients (Madene, Jacquot, Scher, & Desobry, 2006; Muoki, de Kock, & Emmambux, 2012) and vitamin and mineral rich ingredients (Tontisirin, Nantel, & Bhattacharjee, 2002) also improve nutritional properties of cereal‐based diets. The second factor is the ratios in which these ingredients are blended. Studies have shown that optimal blending can optimize the nutritional and sensory quality of complementary foods (Fikiru, Bultosa, Forsido, & Temesgen, 2016; Gebretsadikan, Bultosa, Forsido, & Astatkie, 2015). The third factor is the process conditions used to manufacture the product. There are several food processing techniques used for the production of modified and improved products, and extrusion is among them (Lobato, Anibal, Lazaretti, & Grossmann, 2011).

Extrusion is a unique food processing operation which utilizes high temperature/short time (HTST) and high shear force to produce a product with distinct physical and chemical characteristics. The heat and shear action during extrusion bring about starch gelatinization, protein denaturation, and inactivation of enzymes, microbes, and many antinutritional factors (Lobato et al., 2011). Extrusion of a mixture of starch, protein, and oil may result in the development of color, flavor, and aroma compounds (Muoki, Kinnear, Emmambux, & de Kock, 2015). Extrusion is a technology used to produce ready‐to‐eat (instant) (Mouquet, Salvignol, Van Hoan, Monvois, & Trèche, 2003) and energy‐dense cereal complementary porridge with a viscosity that is palatable to children (Muoki et al., 2012). Generally, extrusion technology helps to produce products of high nutritional and functional quality.

Despite evidence of the application of blending for the formulation of baby foods (Fikiru et al., 2016; Gebretsadikan et al., 2015), there is limited information on the possibility of making extrusion‐cooked nutrient‐rich and tasty complementary food products from blended locally available, nutritious and relatively cheaper crops in Ethiopia. This study aimed to assess the feasibility of developing nutritious and palatable complementary food by optimally blending and extruding oats, soybean, linseed, and a premix.

## 2 MATERIALS AND METHODS

### 2.1 Experimental materials

Ingredients were chosen primarily based on Codex Alimentarius guidelines (Codex Alimentarius Commission, 1991). Accordingly, the blend was composed of a cereal which is commonly used for making complementary food in the study area (oats variety *Sinana One*), a protein‐rich ingredient (soybean variety *Clark 63K*) and a fat‐rich ingredient (linseed variety *Kulumsa One*). Additionally, a premix *of Moringa stenopetala*, fenugreek variety *Chala*, iodized salt, and sugar was also added to supplement micronutrients and improve sensory attributes of composite blends. The samples were packed in airtight polythene bags and stored in a laboratory at room temperature until needed for processing.

### 2.2 Raw material preparation

Oats were cleaned, dehusked, manually sorted, milled (2 mm screen) (Retsch mill, Retsch GmbH, 5657 Haan, West Germany), and passed through a 30‐mesh sieve. The soybeans were cleaned, boiled (30 min), and dehulled. Afterward, the beans were dried at 60°C for about 13 hr, milled into flour, and sieved (0.5 mm sieve) (Gebretsadikan et al., 2015). The linseed was cleaned, lightly roasted (100°C for 15 min) until a nutty flavor was developed, milled, and sieved (0.5‐mm sieve) to get full‐fat linseed flour (Sudha, Begum, & Ramasarma, 2010). Fenugreek seeds were cleaned, roasted (130 ± 5°C for 7 min), ground and passed through standard test sieve (0.5 mm). Fresh mature moringa leaves were plucked, washed, dried under the shade at 20°C for 2 weeks, milled, and sieved (0.5 mm) to produce fine flour (Gebretsadikan et al., 2015). All powder samples were stored at refrigeration temperature (4°C) in an airtight container until used.

### 2.3 Experimental design

A 13‐run constrained D‐optimal mixture experiment was generated using Minitab^®^ (Version 16.0, Minitab, Inc.) software. The constraints used were 55–65 g/100 g for oats, 11–23 g/100 g for soybean and 6–11 g/100 g for linseed. Upper and lower constraints for each ingredient were determined based on data from food composition tables (Souci, Fachmann, & Kraut, 2008) and literature (Mouquet et al., 2003; Nguyen, Rivier, Eymard‐Duvernay, & Trêche, 2010; Pathania, Singh, Sharma, Sharma, & Singla, 2013). The proportion of oats, soybean, and linseed is converted to 85 g/100 g, and the remaining 15 g/100 g was reserved for the premix (9.9 g/100 g sugar, 0.6 g/100 g table salt, three g/100 g moringa, and 1.5 g/100 g fenugreek) in all the runs. The ingredients in the premix and their proportions were determined as follows. Sugar was selected as it improves taste (Mouquet et al., 2003). According to the same author, salt also improves taste and provides iodine. On the other hand, moringa was chosen due to its excellent nutritional profile and the proportion (3%) was chosen based on Gebretsadikan et al. (2015). Fenugreek was included in the premix as mothers in the study area commonly use it for seasoning purposes when preparing complementary foods.

### 2.4 Extrusion

The ingredients were thoroughly mixed for 5 min in a planetary cake mixer (H.LB20/B, Hungary). The extrusion cooking process was performed using a pilot scale co‐rotating twin screw food extruder (model Clextral, BC‐21 N0 194, Firminy, France). The necessary calibration and adjustment of the material feed rate, water flow rate, barrel temperature of the metering section, moisture content of the raw material, and screw speed were performed before the extrusion cooking process. The extruder was operated at a barrel temperature of 130°C, a screw speed of 150 rpm with the feeder delivering feed rate of 5.1 g/min. Feed moisture content in the extruder barrel was 17% (170 g/kg). The extrudate was allowed to cool to 20°C, and afterward dried in a convective oven at 105^o^C for 15 min (Semasaka, Kong, & Hua, 2010), cooled to room temperature, milled, sieved (0.5 mm sieve), and the flour was stored in polythene bags at 4°C.

### 2.5 Gruel preparation

Thirteen gruel samples were prepared from the extruded composite powders. About 50 g of flour was added to 150 ml of water, warmed at 75°C on a thermostatically controlled hot plate, and stirred with a wooden ladle until it attained the desirable pasty consistency similar to the traditional gruel consumed in the study area (Gebretsadikan et al., 2015). After samples had been taken for nutritional analysis, the remaining gruel samples were left to cool to 45°C before sensory evaluation.

### 2.6 Nutrient analysis

Proximate composition of the products was determined on dry matter basis following the Association of Official Analytical Chemists (AOAC) methods: moisture (925.10), crude protein (979.09), crude fiber (962.09), crude fat (920.39), and ash (923.03) (AOAC International, 2005). Total carbohydrate content was calculated by adding the proportion of other ingredients and subtracting from 100. The energy contents of the samples were obtained by multiplying crude protein, crude fat, and carbohydrate by conversion factors of 4, 9, and 4, respectively (Osborne & Voogt, 1978).

Micronutrient content namely calcium (Ca), iron (Fe), and zinc (Zn) were determined using a Flame Atomic Absorption Spectrophotometer (AAS) (Shimadzu, AA‐6800, Japan) following AOAC method 985.35 (AOAC International, 2005). The ß‐carotene content was determined using a UV/Vis spectrophotometer T‐80 (PG Instruments, China) (Biswas, Sahoo, & Chatli, 2011). The micronutrients were quantified against standard solutions of known concentrations.

### 2.7 Sensory analysis

Sensory evaluation for acceptability of gruel was performed by using 50 untrained panels selected from Jimma town, Ethiopia. After orientation, coded sample products were given in a random order to the panelists for evaluation of appearance, aroma, taste, mouthfeel, and overall acceptability. A five‐point hedonic scale (5 = like very much, 4 = like moderately, 3 = neither like nor dislike, 2 = dislike moderately, and 1 = dislike very much) was used (Meilgaard, Carr, & Civille, 2006).

### 2.8 Statistical analysis and optimization

The responses measured from the 13 formulations were analyzed using Minitab^®^, Version 16. Independence, normality, and constant variance assumptions of the error terms were checked. Mixture components were considered as model terms, and mixture regression was selected as a model fitting method. A *p*‐value less than 0.05 was used to designate the statistical significance of association between a response and a term. Positive coefficients for interaction terms were used to indicate that the components in the term act synergistically and vice versa. The “sweet spot” that optimizes the responses was determined using the lower and upper goals for responses which were defined by the researchers (Montgomery, 2013).

## 3 RESULTS AND DISCUSSION

Table 1 summarizes the ANOVA *p*‐values of all the responses, that is proximate composition, mineral content, β‐carotene content, and sensory properties. The quadratic models for protein, carbohydrate, fiber, ash, β‐carotene content, and aroma fit the data very well (*R* ^2^ > 0.90). Likewise, the quadratic models for fat content, taste, and consistency also fit the data well (*R* ^2^ > 0.80). For iron and calcium content, the linear models fit the data better (*p* < 0.01). Conversely, for moisture, energy, and zinc content as well as appearance and overall acceptance both the quadratic and linear models do not fit the data well.

**Table 1:** ANOVA *p*‐values for the quadratic regression model for mixtures of proximate compositions, energy, mineral content, beta‐carotene, and sensory attributes

Source	Proximate composition						Energy	Mineral content			β‐C	Sensory attributes					
	Prot	Fat	CHO	Fiber	Ash	MC		Fe	Zn	Ca		Apr	Aroma	Tas	MF	Con	OA
Linear^a^	0.01	0.26	0.01	0.00	0.01	0.11	0.69	0.01	0.34	0.06	0.00	0.30	0.01	0.02	0.78	0.05	0.06
Quadratic^a^	0.01	0.02	0.00	0.00	0.01	0.13	0.94	0.74	0.94	0.64	0.00	0.09	0.01	0.03	0.24	0.03	0.10
Oats*Soybean	0.06	0.27	0.01	0.67	0.01	0.86	0.85	0.64	0.75	0.95	0.10	0.63	0.01	0.02	0.71	0.04	0.08
Oats*Linseed	0.01	0.18	0.01	0.00	0.01	0.28	0.81	0.87	0.70	0.31	0.00	0.90	0.04	0.01	0.33	0.92	0.04
Soybean*Linseed	0.04	0.02	0.00	0.00	0.00	0.10	0.75	0.57	0.59	0.28	0.00	0.34	0.40	0.01	0.87	0.71	0.06
*R* ^2^ (Adjusted)	0.98	0.97	0.96	0.98	0.91	0.93	0.97	0.99	0.98	0.99	0.94	0.83	0.82	0.83	0.82	0.84	0.61

*Notes*

Apr: appearance; CHO: carbohydrate; Con: consistency; MC: moisture content; MF: mouthfeel; OA: overall acceptability; Prot: protein; *R* ^2^: coefficient of determination; Tas: taste; β‐C: β‐carotene.

a Model fitting method used is mixture regression. Regression *p*‐value less than or equal to 0.05 indicates the model explains variation in the response.

This table shows whether there is a significant relationship between blending ratio and composition and sensory attributes.

### 3.1 Nutritional quality

Table 2 summarizes the proximate composition, calorific value, mineral, and beta‐carotene content of extruded composite flour of oats, soybean, linseed, and premix. There is a statistically significant (*p* < 0.01) relationship between the blend of oats and linseed, soybean and linseed and the protein content. The highest protein content at the highest percentage of soy in the blend might be due to the high protein content of soybean. An increase in the protein content of foods with increased soybean supplement level has been reported (Gebretsadikan et al., 2015). The blending of cereal‐based foods with legumes improves their protein content (Hotz & Gibson, 2007; Onwulata, Smith, Konstance, & Holsinger, 2001). The other protein‐rich ingredients in the premix, moringa leaf powder (24 g/100 g protein content), and fenugreek (23 g/100 g protein content) could have also contributed to the relatively high protein content recorded from the blends (US Department of Agriculture, Agricultural Research Service, & Nutrient Data Laboratory, 2015).

**Table 2:** Proximate composition, calorific value, mineral and beta‐carotene content of extruded composite flour of oats, soybean, linseed, and premix (dry weight basis)

Run	Oats (%)	Soybean (%)	Linseed (%)	MC (g/100 g)	Protein (g/100 g)	Fat (g/100 g)	Fiber (g/100 g)	Ash (g/100 g)	CHO (g/100 g)	Energy (kcal/100 g)	Ca (mg/100 g)	Fe (mg/100 g)	Zn (mg/100 g)	β‐C (μg/g)
1	57.9	19.9	7.2	4.1	19.5	8.5	3.9	2.7	61.4	399.8	116.9	7.5	3.0	15.3
2	57.4	17.9	9.7	4.0	19.4	8.7	3.9	2.8	61.1	400.8	117.6	7.4	3.0	17.3
3	56.0	23.0	6.0	3.2	20.5	8.7	3.9	2.9	60.9	403.7	121.2	7.8	3.0	13.1
4	65.0	11.0	9.0	5.2	18.2	7.1	4.1	2.6	62.7	387.2	101.3	6.6	2.9	19.0
5	62.4	15.4	7.2	4.7	18.7	7.6	4.0	2.7	62.3	392.5	107.8	7.0	3.0	17.8
6	55.0	19.0	11.0	3.2	19.6	9.9	4.0	3.1	60.2	408.2	122.4	7.4	3.0	15.5
7	62.4	13.9	8.7	5.0	18.8	7.5	4.1	2.8	61.9	390.3	107.2	6.9	2.9	18.3
8	57.4	19.9	7.7	4.0	19.9	8.7	3.8	2.9	60.6	400.8	118.7	7.5	3.0	16.9
9	63.0	11.0	11.0	4.6	18.1	7.0	4.3	2.3	63.7	390.1	104.9	6.6	2.9	14.8
10	59.8	16.8	8.3	4.5	19.0	8.5	3.9	2.8	61.4	397.9	113.1	7.2	3.0	17.2
11	55.0	23.0	7.0	3.4	20.6	9.3	3.8	3.1	59.8	405.0	124.4	7.8	3.0	15.0
12	61.4	13.9	9.7	4.3	18.7	7.8	4.1	2.6	62.5	395.0	110.0	6.9	2.9	17.2
13	65.0	14.0	6.0	5.6	18.0	6.8	4.2	2.5	62.9	384.5	102.5	6.8	2.9	16.7

*Notes*

Values are means of 3 analyses.

The sum of the proportion of oats, soybean, and linseed in a run is equal to 85%, and the remaining 15% was reserved for the premix (9.9% sugar, 0.6% salt, 3% moringa, and 1.5% fenugreek) in all the runs.

CHO: carbohydrate; MC: moisture content; β‐C: beta‐carotene.

The association between the blend of soybean and linseed and the fat content is also statistically significant (*p* < 0.05). The fat content increased consistently with increasing soybean and linseed proportions in the blend. A study has reported that the addition of soy flour increased the fat content of wheat flour (Ndife, Abdulraheem, & Zakari, 2011). A 33.7% rise in the fat content of wheat cookies was also reported by increasing the proportion of roasted linseed flour in a blend as linseed contains far higher fat, compared to refined wheat flour (Ganorkar & Jain, 2014). Cooking, whether conventional or extrusion, results in reduced fat recovery probably through the formation of amylose‐lipid complexes (Muoki et al., 2012).

The current study found that the blend of oats and soybean, oats and linseed, and soybean and linseed have a statistically significant (*p* < 0.01) relationship with the carbohydrate content. Oats contain nearly twice the carbohydrate content as compared to soybean or linseed (Souci et al., 2008). Extrusion cooking might have also contributed to the reduction in total carbohydrate content through sugar losses (conversion of sucrose into glucose and fructose) (Singh, Gamlath, & Wakeling, 2007), and gelatinization and partial dextrinization of starch (Mouquet et al., 2003).

A significant (*p* < 0.01) association was observed between the blend of oats and linseed, soybean and linseed and the fiber content. Oats and linseed, and soybean and linseed act antagonistically. All the three main ingredients naturally have high fiber content. Nevertheless, dehulling during sample preparation removes the hulls, which contain much fiber (Kikafunda, Abenakyo, & Lukwago, 2006).

There is a statistically significant (*p* < 0.01) relationship between the blend of oats and soybean, oats and linseed, soybean and linseed and the ash content. Oats and soybean act antagonistically while oats and linseed, and soybean and linseed act synergistically. Soybean has a higher ash content than oats (Souci et al., 2008). A similar result where ash contents of blended products increased as the proportion of soybean flour increased has been reported (Okoye, Nkwocha, & Ogbonnaya, 2008). On the contrary, the ash content of wheat flour was increased by the incorporation of oilseed meal (Jan, Sattar, Mehmood, & Ali, 2000).

A significant (*p* < 0.01) association was observed between the linear terms and the iron content. The iron content of the blend was directly associated with the soybean supplement levels. Other studies also have indicated that a higher level of soy in composite flour is associated with a higher level of iron (Bolarinwa, Olaniyan, Adebayo, & Ademola, 2015; Gebretsadikan et al., 2015). The presence of iron‐rich ingredients like moringa leaf powder (32.5 mg/100 g) and fenugreek (35.53 mg/100 g) in the premix could have also increased the iron content in the blends (Gebretsadikan et al., 2015).

Similarly, a significant (*p* < 0.01) association was observed between the linear terms and the calcium content. Gruel's calcium content increased significantly with increase in soybean supplement levels. Other researchers have also reported an increase in calcium content of composites with an increase in soybean supplementation (Bolarinwa et al., 2015). Furthermore, oilseed flours (Jan et al., 2000) and moringa leaf powder (Gebretsadikan et al., 2015) also contain an appreciable quantity of minerals and upon addition can increase in the calcium contents of blends.

The association between the blend of oats and linseed, soybean and linseed, and the β‐carotene content is statistically significant (*p* < 0.01). Oats and linseed, and soybean and linseed act synergistically. Carotenoids are commonly found mixed with different macromolecules in the food matrix, and thermal processing helps to efficiently release them from these matrices (Hotz & Gibson, 2007). The high ß‐carotene content of the composite extruded flour could also be associated with the 3% moringa added in the formulation. Moringa leaf powder supplementation is reported to be associated with increased ß‐carotene content in the wheat flour (Sengev, Abu, & Gernah, 2013).

### 3.2 Sensory quality

Table 3 summarizes sensory scores of porridges made from extruded composite flour of oats, soybean, linseed, and premix. The current study found that the blend of oats and soybean, and oats and linseed have a statistically significant (*p* < 0.01) relationship with the aroma. Oats and soybean, and oats and linseed act antagonistically. That is, the mean aroma value is less than the value would be obtained by calculating the simple mean of the aroma for each pure mixture. Usually, heating during extrusion cooking releases flavor in soybean and linseed (Ojinnaka, Ebinyasi, Ihemeje, & Okorie, 2013). However, the blending of these ingredients with oat flour somehow limited the release of aroma components upon cooking. The main volatiles occurring in low‐temperature (120°C) and high‐moisture (22%) extrusion are compounds associated with lipid degradation, with few compounds derived from the Maillard reaction (Bredie, Mottram, & Guy, 1998).

**Table 3:** Sensory scores of porridges made from extruded composite flour of oats, soybean, linseed, and premix

Run number	Oats (%)	Soybean (%)	Linseed (%)	Premix^a^ (%)	Appearance	Aroma	Taste	Mouthfeel	Consistency	Overall acceptance
1	57.9	19.9	7.2	15.0	3.4	3.6	3.5	3.7	3.7	3.6
2	57.4	17.9	9.7	15.0	3.4	3.7	3.6	3.7	3.7	3.8
3	56.0	23.0	6.0	15.0	3.3	3.6	3.6	3.6	3.8	3.7
4	65.0	11.0	9.0	15.0	3.2	3.7	3.8	3.5	4.0	4.0
5	62.4	15.4	7.2	15.0	3.5	3.6	3.6	3.5	3.8	3.6
6	55.0	19.0	11.0	15.0	3.1	3.8	3.5	3.7	3.7	3.6
7	62.4	13.9	8.7	15.0	3.4	3.6	3.7	3.6	3.8	3.9
8	57.4	19.9	7.7	15.0	3.2	3.6	3.5	3.7	3.6	4.0
9	63.0	11.0	11.0	15.0	3.0	3.7	3.5	3.7	3.9	3.8
10	59.8	16.8	8.3	15.0	3.5	3.6	3.6	3.6	3.6	3.8
11	55.0	23.0	7.0	15.0	3.4	3.7	3.6	3.8	3.8	3.8
12	61.4	13.9	9.7	15.0	3.3	3.6	3.6	3.6	3.9	3.8
13	65.0	14.0	6.0	15.0	3.7	3.8	3.6	3.4	3.8	3.8

*Notes*

Values are means of 50 rankings on a five‐point hedonic scale.

a Premix is 9.9% sugar, 0.6% salt, 3% moringa, and 1.5% fenugreek.

A significant (*p* < 0.01) association was observed between the oats and soybean, oats and linseed, soybean and linseed and the taste. Oats and soybean act antagonistically whereas oats and linseed, and soybean and linseed act synergistically. Mainly, linseed is a contributor toward high taste ranking. Beany taste of soy (related to lipoxygenases) may not have been masked by oats, which in turn significantly reduces the taste ranking of products (Bott & Chambers, 2006). On the other hand, the big nutty flavor imparted by linseed has blended well with oats taste as well as masked the beany flavor of soybean. Besides the blending of ingredients, extrusion processing is also reported to be used to make products with no or minimal bean flavor (Nyombaire, Siddiq, & Dolan, 2011). Dextrinization (a nonenzymatic browning and chemical process which breaks down starch into dextrin's [disaccharides]) and starch breakdown take place during the extrusion process thereby enhancing the taste of gruel prepared from extruded flour (Mensah & Tomkins, 2003).

There is a statistically significant (*p* < 0.01) relationship between the blend of oats and soybean and the consistency. Consistency is a major factor determining the volume and energy density of starch‐based diets and should be given due emphasis especially when formulating starch‐based products for preschool‐age children. Protein–protein and protein–carbohydrate interactions were reported to influence gruel consistency, with soy protein significantly increasing viscosity (Hellstrom et al., 1981).

### 3.3 Gruel optimal mixture compositions

**Figure 1:**
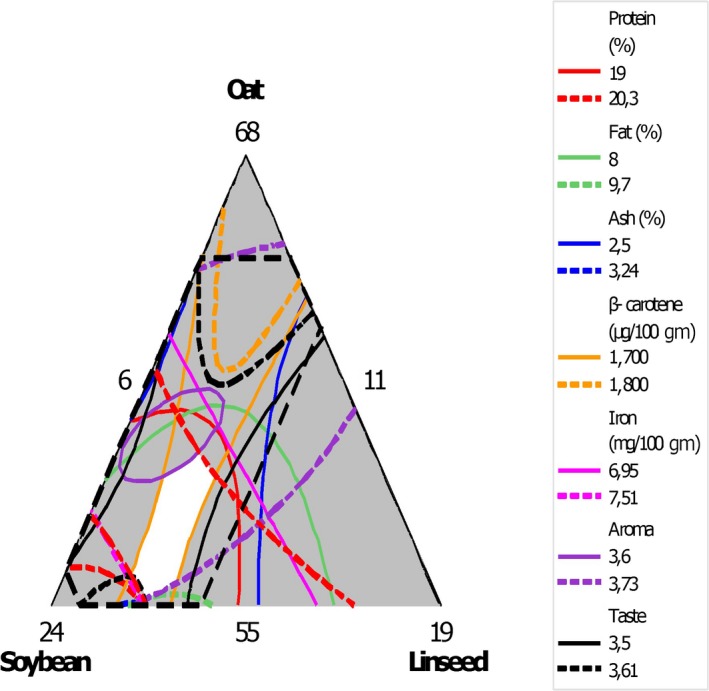
Overlaid contour plots that show the sweet spot. Notes: The white area shows the “sweet spot” that optimizes the response variables listed in the respective legends

For optimization purposes, only responses that showed a statistically significant relationship with the blend components were considered. Accordingly, the researchers defined the following lower and upper goals for responses: protein (20–20.3 g/100 g), fat (9.6–9.7 g/100 g), ash (2.5–3.24 g/100 g), carbohydrate (59.4–60 g/100 g), ß‐carotene (1,740–1,740.2 μg/100 g), calcium (123–123.1 mg/100 g), iron (7.5–7.51 mg/100 g), aroma (3.6–3.73), taste (3.5–3.61), and consistency (3.6–3.7). The superimposed contour plots are presented in Figure 1, and the white area shows the “sweet spot” where the blending ratio of ingredients results in an optimal response.

Overall optimization of both nutritional and sensory qualities showed that blend ratio of 55.0 g/100 g oats, 21.0 g/100 g soybean, and 9.0 g/100 g linseed gave optimal compositions of 20.3 g/100 g protein, 9.8 g/100 g fat, 3.2 g/100 g ash, 59.4 g/100 g carbohydrates, 1,740.2 g/100 g ß‐carotene, 123.2 mg/100 g calcium, and 7.52 mg/100 g iron content. Additionally, the aroma, taste, and consistency ranking of this blend were 3.7, 3.6, and 3.7, respectively. The response optimizer showed that the goals we defined for the responses were achievable (composite desirability of 0.9992.

## 4 CONCLUSIONS

The results showed that it is feasible to develop a nutritious complementary food with acceptable sensory properties by compositing and extrusion cooking of oats, soybean, linseed, and premix. The optimal mixture of 55.0 g/100 g oats, 21.0 g/100 g soybean, and 9.0 g/100 g linseed flour with added 15 g/100 g premixes induced significant improvement in the composite flour's nutritional quality and gruel's sensory attributes which can contribute to the fight against child undernutrition. Small and medium food enterprises can make use of these results as a starting point for commercialization of the developed product.

## CONFLICT OF INTEREST

The authors declare that they do not have any conflict of interest.

## ETHICAL STATEMENTS

This study was approved by the Institutional Review Board of Jimma University. All procedures involving human participants were in accordance with the ethical standards of the 1964 Helsinki declaration and its later amendments. Written informed consent was obtained from all study participants.

## References

[fsn3940-bib-0001] AOAC International (2005). Official methods of analysis of AOAC International, 18th ed Gaithersburg, MD: AOAC International.

[fsn3940-bib-0002] Biswas, A. K. , Sahoo, J. , & Chatli, M. K. (2011). A simple UV‐Vis spectrophotometric method for determination of β‐carotene content in raw carrot, sweet potato and supplemented chicken meat nuggets. LWT ‐ Food Science and Technology, 44(8), 1809–1813. 10.1016/j.lwt.2011.03.017

[fsn3940-bib-0003] Bolarinwa, I. F. , Olaniyan, S. A. , Adebayo, L. O. , & Ademola, A. A. (2015). Malted sorghum‐Soy composite flour: Preparation, chemical and physico‐chemical properties. Journal of Food Processing & Technology, 6(8), 1.

[fsn3940-bib-0004] Bott, L. , & Chambers, E. (2006). Sensory characteristics of combinations of chemicals potentially associated with beany aroma in foods. Journal of Sensory Studies, 21(3), 308–321. 10.1111/j.1745-459X.2006.00067.x

[fsn3940-bib-0005] Bredie, W. L. P. , Mottram, D. S. , & Guy, R. C. E. (1998). Aroma Volatiles Generated during Extrusion Cooking of Maize Flour. Journal of Agricultural and Food Chemistry, 46(4), 1479–1487. 10.1021/jf9708857

[fsn3940-bib-0006] Central Statistical Agency . (2012). Household Consumption Expenditure Survey 2010/11 ‐ Statistical Report. Addis Ababa. Retrieved from http://catalog.ihsn.org/index.php/catalog/3123/download/46157

[fsn3940-bib-0007] Central Statistical Agency [Ethiopia], & ICF International . (2012). Ethiopia Demographic and Health Survey 2011. Retrieved from http://www.usaid.gov/sites/default/files/documents/1860/Demographic Health Survey 2011 Ethiopia Final Report.pdf

[fsn3940-bib-0008] Codex Alimentarius Commission (1991). Guidelines for formulated supplementary foods for older infants and young children. Rome, Italy: Joint FAO/WHO Food Standards Programme Codex Alimentarius Commission.

[fsn3940-bib-0009] Egounlety, M. (2002). Production of legume‐fortified weaning foods. Food Research International, 35(2–3), 233–237. 10.1016/S0963-9969(01)00190-9

[fsn3940-bib-0010] Fikiru, O. , Bultosa, G. , Forsido, S. F. , & Temesgen, M. (2016). Nutritional quality and sensory acceptability of complementary food blended from maize (Zea mays), roasted pea (Pisum sativum), and malted barley (Hordium vulgare). Food Science & Nutrition, 5(2), 173–181. 28265352 10.1002/fsn3.376 PMC5332271

[fsn3940-bib-0011] Ganorkar, P. M. , & Jain, R. K. (2014). Effect of flaxseed incorporation on physical, sensorial, textural and chemical attributes of cookies. International Food Research Journal, 21(4), 1515–1521.

[fsn3940-bib-0012] Gebretsadikan, T. M. , Bultosa, G. , Forsido, S. F. , & Astatkie, T. (2015). Nutritional quality and acceptability of sweet potato‐soybean‐moringa composite porridge. Nutrition & Food Science, 46(5), 845–858. Retrieved from http://www.emeraldinsight.com/doi/abs/10.1108/NFS-05-2015-0048

[fsn3940-bib-0013] Hellstrom, A. , Hermansson, A.‐M. , Karlsson, A. , Ljungqvist, B. , Mellander, O. , & Svanberg, U. (1981). Dietary bulk as a limiting factor for nutrient intake—with special reference to the feeding of pre‐school children. II. Consistency as related to dietary bulk–a model study. Journal of Tropical Pediatrics, 27(3), 127–135. 10.1093/tropej/27.3.127

[fsn3940-bib-0014] Hotz, C. , & Gibson, R. S. (2007). Traditional food‐processing and preparation practices to enhance the bioavailability of micronutrients in plant‐based diets. Journal of Nutrition, 137(4), 1097–1100. Retrieved from http://jn.nutrition.org/content/137/4/1097.short 17374686 10.1093/jn/137.4.1097

[fsn3940-bib-0015] Jan, M. , Sattar, A. , Mehmood, F. , & Ali, Y. (2000). Chemical and technological evaluation of fortified wheat bread (Chapati) with oilseed flours. Sarhad Journal of Agriculture, 16(1), 85–88.

[fsn3940-bib-0016] Kikafunda, J. K. , Abenakyo, L. , & Lukwago, F. B. (2006). Nutritional and sensory properties of high energy/nutrient dense composite flour porridges from germinated maize and roasted beans for child‐weaning in developing countries: A case for Uganda. Ecology of Food and Nutrition, 45(4), 279–294. 10.1080/03670240600846344

[fsn3940-bib-0017] Lobato, L. P. , Anibal, D. , Lazaretti, M. M. , & Grossmann, M. V. E. (2011). Extruded puffed functional ingredient with oat bran and soy flour. LWT ‐ Food Science and Technology, 44(4), 933–939. 10.1016/j.lwt.2010.11.013

[fsn3940-bib-0018] Madene, A. , Jacquot, M. , Scher, J. , & Desobry, S. (2006). Flavour encapsulation and controlled release ‐ a review. International Journal of Food Science and Technology, 41(1), 1–21. 10.1111/j.1365-2621.2005.00980.x

[fsn3940-bib-0019] Meilgaard, M. C. , Carr, B. T. , & Civille, G. V. (2006). Sensory Evaluation Techniques, 4th ed Boca Raton, FL: CRC Press 10.1201/b16452

[fsn3940-bib-0020] Mensah, P. , & Tomkins, A. (2003). Household‐level technologies to improve the availability and preparation of adequate and safe complementary foods. Food and Nutrition Bulletin, 24(1), 104–125. 10.1177/156482650302400106 12664529

[fsn3940-bib-0021] Montgomery, D. C. (2013). Design and Analysis of Experiments, 8th ed New York, NY: Wiley.

[fsn3940-bib-0022] Mouquet, C. , Salvignol, B. , Van Hoan, N. , Monvois, J. , & Trèche, S. (2003). Ability of a “very low‐cost extruder” to produce instant infant flours at a small scale in Vietnam. Food Chemistry, 82(2), 249–255. 10.1016/S0308-8146(02)00545-9

[fsn3940-bib-0023] Muhimbula, H. S. , Issa‐Zacharia, A. , & Kinabo, J. (2011). Formulation and sensory evaluation of complementary foods from local, cheap and readily available cereals and legumes in Iringa. Tanzania. African Journal of Food Science, 5(1), 26–31.

[fsn3940-bib-0024] Muoki, P. N. , de Kock, H. L. , & Emmambux, M. N. (2012). Effect of soy flour addition and heat‐processing method on nutritional quality and consumer acceptability of cassava complementary porridges. Journal of the Science of Food and Agriculture, 92(8), 1771–1779. 10.1002/jsfa.5545 22246672

[fsn3940-bib-0025] Muoki, P. N. , Kinnear, M. , Emmambux, M. N. , & de Kock, H. L. (2015). Effect of the addition of soy flour on sensory quality of extrusion and conventionally cooked cassava complementary porridges. Journal of the Science of Food and Agriculture, 95(4), 730–738. 10.1002/jsfa.6820 25042021

[fsn3940-bib-0026] Muteki, K. , MacGregor, J. F. , & Ueda, T. (2007). Mixture designs and models for the simultaneous selection of ingredients and their ratios. Chemometrics and Intelligent Laboratory Systems, 86(1), 17–25. 10.1016/j.chemolab.2006.08.003

[fsn3940-bib-0027] Ndife, J. , Abdulraheem, L. O. , & Zakari, U. M. (2011). Evaluation of the nutritional and sensory quality of functional breads produced from whole wheat and soya bean flour blends. African Journal of Food Science, 5(8), 466–472.

[fsn3940-bib-0028] Nguyen, V. H. , Rivier, C. M. , Eymard‐Duvernay, S. , & Trêche, S. (2010). Effect of extrusion cooking and amylase addition to gruels to increase energy density and nutrient intakes by Vietnamese infants. Asia Pacific Journal of Clinical Nutrition, 19(3), 308–315. 20805073

[fsn3940-bib-0029] Nyombaire, G. , Siddiq, M. , & Dolan, K. D. (2011). Physico‐chemical and sensory quality of extruded light red kidney bean (Phaseolus vulgaris L.) porridge. LWT ‐ Food Science and Technology, 44(7), 1597–1602. 10.1016/j.lwt.2011.02.016

[fsn3940-bib-0030] Ojinnaka, M. C. , Ebinyasi, C. S. , Ihemeje, A. , & Okorie, S. U. (2013). Nutritional evaluation of complementary food gruels formulated from blends of soybean flour and ginger modified cocoyam starch. Advance Journal of Food Science and Technology, 5(10), 1325–1330. 10.19026/ajfst.5.3105

[fsn3940-bib-0031] Okoye, J. I. , Nkwocha, A. C. , & Ogbonnaya, A. E. (2008). Production, proximate composition and consumer acceptability of biscuits from wheat/soybean flour blends. Continental Journal of Food Science and Technology, 2, 6–13.

[fsn3940-bib-0032] Onwulata, C. I. , Smith, P. W. , Konstance, R. P. , & Holsinger, V. H. (2001). Incorporation of whey products in extruded corn, potato or rice snacks. Food Research International, 34(8), 679–687. 10.1016/S0963-9969(01)00088-6

[fsn3940-bib-0033] Osborne, D. R. , & Voogt, P. (1978). The analysis of nutrients in foods. London, UK: Academic Press Inc.

[fsn3940-bib-0034] Pathania, S. , Singh, B. , Sharma, S. , Sharma, A. , & Singla, S. (2013). Optimization of extrusion processing conditions for preparation of an instant grain base for use in weaning foods. Optimization, 3(3), 1040–1049.

[fsn3940-bib-0035] Semasaka, C. , Kong, X. Z. , & Hua, Y. (2010). Optimization of extrusion on blend flour composed of corn, millet and soybean. Pakistan Journal of Nutrition, 9(3), 291–297.

[fsn3940-bib-0036] Sengev, A. I. , Abu, J. O. , & Gernah, D. I. (2013). Effect of Moringa oleifera leaf powder supplementation on some quality characteristics of wheat bread. Food and Nutrition Sciences, 4(3), 270 10.4236/fns.2013.43036

[fsn3940-bib-0037] Singh, S. , Gamlath, S. , & Wakeling, L. (2007). Nutritional aspects of food extrusion: A review. International Journal of Food Science & Technology, 42(8), 916–929. 10.1111/j.1365-2621.2006.01309.x

[fsn3940-bib-0038] Souci, S. W. , Fachmann, W. , & Kraut, H. (2008). Food Composition and Nutrition Tables, 7th ed Stuttgart, Germany: MedPharm.

[fsn3940-bib-0039] Sudha, M. L. , Begum, K. , & Ramasarma, P. R. (2010). Nutritional characteristics of linseed/flaxseed (Linum usitatissimum) and its application in muffin making. Journal of Texture Studies, 41(4), 563–578.

[fsn3940-bib-0040] Tontisirin, K. , Nantel, G. , & Bhattacharjee, L. (2002). Food‐based strategies to meet the challenges of micronutrient malnutrition in the developing world. Proceedings of the Nutrition Society, 61(02), 243–250. 10.1079/PNS2002155 12133206

[fsn3940-bib-0041] US Department of Agriculture, Agricultural Research Service, & Nutrient Data Laboratory . USDA National Nutrient Database for Standard Reference, Release 28. Version Current (2015). http://www.ars.usda.gov/ba/bhnrc/ndl Accessed 12.08.16.

